# Survival outcomes of autologous breast reconstruction after mastectomy: A matched case-control study

**DOI:** 10.3389/fonc.2022.1022925

**Published:** 2023-01-06

**Authors:** Shang Wu, Xindi Ma, Xiangmei Zhang, Chao Yang, Yubin Wang, Yunjiang Liu

**Affiliations:** ^1^ Breast Center, Fourth Hospital of Hebei Medical University, Shijiazhuang, China; ^2^ Hebei Provincial Key Laboratory of Tumor Microenvironment and Drug Resistance, Hebei Medical University, Shijiazhuang, China

**Keywords:** breast cancer, autologous breast reconstruction, prognosis, PSM, SEER

## Abstract

**Background:**

Due to the lack of strong evidence-based medical evidence, the relationship between autologous breast reconstruction (ABR) after mastectomy and long-term prognosis is unclear. This study aims to explore if ABR after mastectomy is associated with the prognosis of breast cancer (BC) patients based on the data from the Surveillance, Epidemiology, and End Results (SEER) database.

**Methods:**

We collected data for all cases diagnosed with BC who underwent or did not undergo ABR after mastectomy from 2010-2015 in the SEER database. The primary outcome of our study was overall survival (OS) and cancer specific survival (CSS). The Propensity Score-Matched (PSM) analysis was used to eliminate the effects of non-random statistics, setting the caliper as 0.0001 to balance the baseline variables within the groups. Chi-square test, Kaplan-Meier method, univariate and multivariate cox regression analysis were used to analyze the data and subgroup analysis was performed to find the subgroups of people who might benefit from ABR.

**Result:**

Of 27893 eligible patients, 11038 patients were matched. The cohort consisted of 5519 (50%) ABR patients and 5519 (50%) non-ABR patients after PSM. After PSM, on multivariate cox regression analysis, ABR still exerted a significant influence on the OS (hazard ratio (HR), 0.83, *P*< 0.05). However, no statistical difference was shown on CSS (HR, 0.93, *P* = 0.31). Kaplan-Meier survival analysis showed ABR group had better OS (*P* = 0.001), but similar CSS (*P* = 0.174) between ARB and mastectomy groups. Subgroup analysis showed that after matching, those with 50-59 years old, earlier stages of disease, without a marital partner and living in urban areas had better OS after ABR.

**Conclusions:**

ABR after mastectomy was associated with better OS, but not affect CSS.

## Introduction

Breast cancer (BC) is still the most common cancer in women in the world, and in America it represents 30% of the female cancer (1). The incidence of breast cancer and the number of people with the disease are increasing, so the population with a history of breast cancer is a large group. About 23-33% women with breast cancer need mastectomy, causing severe physical and psychological trauma (2, 3). With the continuous improvement of surgical techniques and the improvement of patients’ demand for postoperative quality of life, more and more patients begin to choose breast reconstruction, either immediate reconstruction or late reconstruction after mastectomy (4). Breast reconstruction can be performed using autologous tissue, implants or a combination of these. Autologous tissue reconstruction (ABR) could be divided into latissimus dorsi flap, pedicle transverse rectus abdominis myocutaneous (pTRAM) flap, muscle-sparing free-pTRAM flap, deep inferior epigastric perforator flap, etc. There is an increasing popularity of ABR as a part of therapeutic strategy for breast cancer (5). Because ABR is associated with favorable aesthetic outcomes, less psychological burden, suitability and durability (6, 7). Some articles think patients may harbor dormant micro-metastases at the time of the autologous breast reconstruction (8–16). However, most of the literatures are old, and now there are many treatments available to reduce patients’ risk for relapse. Recent literature suggests that there is no effect of autologous breast reconstruction on distant relapse rate and thus that autologous breast reconstruction is an oncological safe procedure (17, 18), but the sample size of the studies in the article was small. Due to the lack of strong evidence-based medical evidence, the relationship between ABR after mastectomy and long-term prognosis is unclear.

The aim of this study was to evaluate the long-term prognosis of breast cancer patients who underwent ABR after mastectomy versus those who did not, based on a large sample size in the SEER database.

## Methods

Clinical data on BC with surgery were retrieved by using the SEER*Stat version 8.4.0.1. Since human epidermal growth factor receptor 2 (HER2) related data were only included in the database after 2010, we only included patients from 2010 to 2015. This population-based database collects information on cancer patients in 17 registries, representing nearly 30% of the US population (www.seer.cancer.gov). In the current study, we included clinicopathological data, sociological data and treatment data.

Patients receiving mastectomy including simple mastectomy and modified radical with or without removal of uninvolved contralateral breast and received ABR procedure after mastectomy were considered eligible for inclusion. Exclusion criteria included patients with no explicit type of basic characteristics and survival data missing/unknown, second other tumors, male BC patients and clinical stage IV at diagnosis. The pathological type of all patients was breast infiltrating ductal carcinoma (histologic codes 8500 of ICD-O-3). Since this study used registry data, this study was exempted by the ethics committee of the Forth Hospital of Hebei Medical University. The methods were based on approved guidelines (19).

In total, 27893 patients were included in this study, including BC patients receiving reconstruction (N = 5912) or not (N = 21981) after mastectomy. As local recurrence data are unavailable in the SEER database, the primary outcome of our study was overall survival (OS) and cancer specific survival (CSS). We defined OS was determined based on the date of diagnosis to death from any cause. The CSS from the time of initial diagnosis to the time of cancer-related death.

The clinicopathological characteristics of BC patients before and after propensity score matching (PSM) were included in the analysis (**Table 1**): clinical age, race, tumor grade, radiotherapy, chemotherapy, T and N stage according to the American Joint Committee on Cancer (AJCC) staging system sixth edition (Breast-Adjusted AJCC 6th Stage), marital status, income and living region. Breast subtype has four types, including hormone receptor-positive (HR+)/human epidermal growth factor receptor 2 negative (HER2-); HR+/HER2+; HR-/HER2+; HR-/HER2-.

**Table 1:** The clinicopathological characteristics of BC patients before and after PSM.

	Before PSM			After PSM		
	M	ABR	*P*	M	ABR	*P*
**Variables**	N=21981	N=5912		N=5519	N=5519	
**Clinical–Age**			<0.001			0.954
20-49	10633 (48.4%)	3476 (58.8%)		3188 (57.8%)	3192 (57.8%)	
50-59	11348 (51.6%)	2436 (41.2%)		2331 (42.2%)	2327 (42.2%)	
**Race**			<0.001			0.76
White	15742 (71.6%)	4542 (76.8%)		4363 (79.1%)	4341 (78.7%)	
Black	2915 (13.3%)	753 (12.7%)		576 (10.4%)	600 (10.9%)	
Other/unknown	3324 (15.1%)	617 (10.4%)		580 (10.5%)	578 (10.5%)	
**Marital-**status			<0.001			0.934
No	8426 (38.3%)	1784 (30.2%)		1639 (29.7%)	1634 (29.6%)	
Yes	13555 (61.7%)	4128 (69.8%)		3880 (70.3%)	3885 (70.4%)	
**Tumor-Grade**			<0.001			0.991
I/II	10354 (47.1%)	3084 (52.2%)		2942 (53.3%)	2937 (53.2%)	
III/IV	11035 (50.2%)	2664 (45.1%)		2496 (45.2%)	2502 (45.3%)	
Unknown	592 (2.69%)	164 (2.77%)		81 (1.47%)	80 (1.45%)	
**T-TNM**			<0.001			0.921
1	9124 (41.5%)	3300 (55.8%)		3079 (55.8%)	3088 (56.0%)	
2	8989 (40.9%)	2078 (35.1%)		1980 (35.9%)	1973 (35.7%)	
3	2581 (11.7%)	441 (7.46%)		379 (6.87%)	385 (6.98%)	
4	1287 (5.86%)	93 (1.57%)		81 (1.47%)	73 (1.32%)	
**N-TNM**			<0.001			0.972
0	10496 (47.8%)	3649 (61.7%)		3431 (62.2%)	3437 (62.3%)	
1	7664 (34.9%)	1690 (28.6%)		1598 (29.0%)	1585 (28.7%)	
2	2382 (10.8%)	395 (6.68%)		336 (6.09%)	346 (6.27%)	
3	1439 (6.55%)	178 (3.01%)		154 (2.79%)	151 (2.74%)	
**M**olecular subtype			<0.001			0.879
HR+/HER2-	12737 (57.9%)	3745 (63.3%)		3605 (65.3%)	3586 (65.0%)	
HR+/HER2+	3516 (16.0%)	921 (15.6%)		804 (14.6%)	824 (14.9%)	
HR-/HER2+	1856 (8.44%)	408 (6.90%)		329 (5.96%)	342 (6.20%)	
HR-/HER2-	3872 (17.6%)	838 (14.2%)		781 (14.2%)	767 (13.9%)	
**Treatment- Radiotherapy**			<0.001			0.964
No/unknown	14312 (65.1%)	4546 (76.9%)		4272 (77.4%)	4269 (77.4%)	
Yes	7669 (34.9%)	1366 (23.1%)		1247 (22.6%)	1250 (22.6%)	
**Treatment- Chemotherapy**			<0.001			0.844
No/unknown	6293 (28.6%)	2226 (37.7%)		2097 (38.0%)	2086 (37.8%)	
Yes	15688 (71.4%)	3686 (62.3%)		3422 (62.0%)	3433 (62.2%)	
**Income**			<0.001			0.878
<60000$	8018 (36.5%)	1498 (25.3%)		1382 (25.0%)	1374 (24.9%)	
≥60000$	13963 (63.5%)	4414 (74.7%)		4137 (75.0%)	4145 (75.1%)	
**Region**			<0.001			0.694
Urban	19426 (88.4%)	5582 (94.4%)		5249 (95.1%)	5239 (94.9%)	
Rural	2555 (11.6%)	330 (5.58%)		270 (4.89%)	280 (5.07%)	

N, number; M, mastectomy; ABR, autologous breast reconstruction; OS, overall survival; CSS, cancer specific survival; HR, hormone receptor; HER2, human epidermal growth factor receptor 2.

The baseline characteristics of included patients was simply described by using frequencies and percentages. Chi-square test was used for statistical analysis of differences between groups. We performed PSM by R version 4.1.1 software, setting the caliper as 0.0001 to balance the baseline variables which are shown in **Table 1** including 11 variables. Univariate cox regression analysis was performed to explore the influence of surgical methods on OS and CSS survival and 11 variables shown in **Table 1** were used to conduct multivariate cox regression analysis to explore the influence of surgical methods on OS and CSS before and after PSM. Cox regression analysis was used for subgroups analysis of interest. OS and CSS were assessed according to whether or not patients received ABR through Kaplan-Meier analysis. The log-rank test was performed to determine statistical significance. A corresponding 95% confidence interval (CI) was calculated, and a two-tailed *P*-value < 0.05 was considered statistically significant. Statistical analyses were conducted through R version 4.1.1 software.

## Result

We analyzed the data of 27893 patients from the SEER database in 2010–2015. The group was stratified by whether ABR was performed after mastectomy (**Table 1**), including patients with breast cancer receiving ABR (N = 5912) or not (N = 21981). After PSM, a total of 11038 patients (ABR 5519 vs. non-ABR 5519) were matched, and the covariates were properly balanced between the two groups (*P*> 0.05). Pre-matching results showed a that patients submitted do reconstruction as they are younger, white race, married, lower grade tumors, lower clinical T-TNM and N-TNM stage, HR+/HER2-, having more income and living more in the urban, with lower proportion of patients submitted to radiation therapy, chemotherapy (all *P*< 0.05). Post-matching results did not show significant difference between the groups.

**Figure 1:**
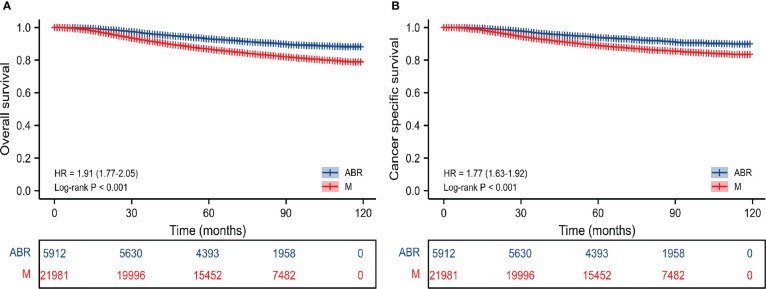
Kaplan-Meier survival curves of survival analysis on OS **(A)** and CSS **(B)** before PSM in the entire cohort. M, mastectomy; ABR, autologous breast reconstruction; OS, overall survival; CSS, cancer specific survival.

In the entire cohort, OS and CSS were compared in 27893 BC patients who underwent ABR after mastectomy or not. The 5-and 8-year OS rates were 93.4% and 91.3% for ABR group compared with 87.3% and 84.1% for mastectomy group. The 5-and 8-year CSS rates were 94.2% and 92.5% for patients undergoing ABR, as compared with 89.6% and 87.2% for patients undergoing mastectomy (**Table 2**). In Kaplan-Meier survival analysis (**Figure 1**), ABR group had better OS (*P*< 0.001) and CSS (*P*<0.001) compared with mastectomy group. The effect of surgery treatment on prognosis was explored on the basis of a univariate cox regression analysis, which suggested that the OS (hazard ratio (HR), 0.52; 95%CI 0.48-0.57, *P*< 0.05) and CSS (HR, 0.56; 95%CI 0.51-0.62, *P*< 0.05) benefit of ABR remained significant and multivariate cox regression analysis also showed that ABR was associated with better OS (HR, 0.74; 95%CI 0.67-0.81, *P*< 0.05) and CSS (HR, 0.83; 95%CI 0.75-0.92, *P*< 0.05). (**Table 3**).

**Table 2:** The 5-and 8-year OS and CSS rates of BC patients.

The entire cohort before PSM					
	M (N=21981)	ABR (N=5912)		M (N=21981)	ABR (N=5912)
5-year OS	19199 (87.3%)	5519 (93.4%)	5-year CSS	19692 (89.6%)	5569 (94.2%)
8-year OS	18485 (84.1%)	5396 (91.3%)	8-year CSS	19175 (87.2%)	5467 (92.5%)
**The entire cohort after PSM**					
	**M (N=5519)**	**ABR (N=5519)**		**M (N=5519)**	**ABR (N=5519)**
5-year OS	5077 (92.0%)	5169 (93.7%)	5-year CSS	5165 (93.6%)	5216 (94.5%)
8-year OS	4947 (89.6%)	5053 (91.6%)	8-year CSS	5081 (92.1%)	5120 (92.8%)

N, number; M, mastectomy; ABR, autologous breast reconstruction; OS, overall survival; CSS, cancer specific survival.

**Table 3:** Univariate and multivariate cox regression analysis of OS and CSS.

	N	OS		CSS	
		HR (95%CI)	*P*	HR (95%CI)	*P*
Univariate cox regression analysis					
**The entire cohort before PSM**	27893				
Mastectomy	21981	1 (Reference)		1 (Reference)	
ABR	5912	0.52 (0.48-0.57)	<0.05	0.56 (0.51-0.62)	<0.05
**The entire cohort after PSM**	11038				
Mastectomy	5519	1 (Reference)		1 (Reference)	
ABR	5519	0.82 (0.72-0.92)	<0.05	0.91 (0.80-1.04)	0.17
Multivariate cox regression analysis					
**The entire cohort before PSM**	27893				
Mastectomy	21981	1 (Reference)		1 (Reference)	
ABR	5912	0.74 (0.67-0.81)	<0.05	0.83 (0.75-0.92)	<0.05
**The entire cohort after PSM**	11038				
Mastectomy	5519	1 (Reference)		1 (Reference)	
ABR	5519	0.83 (0.74-0.94)	<0.05	0.93 (0.82-1.07)	0.31

N, number; M, mastectomy; ABR, autologous breast reconstruction; OS, overall survival; CSS, cancer specific survival.

**Figure 2:**
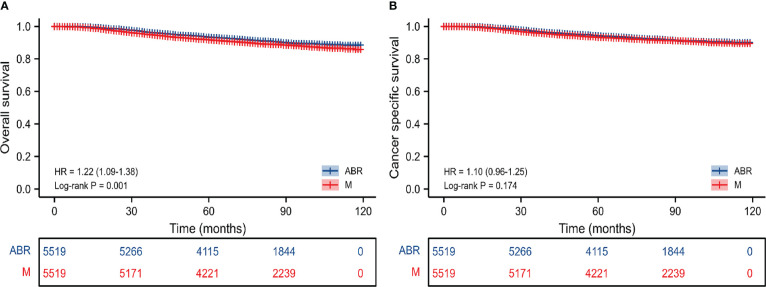
Kaplan-Meier survival curves of survival analysis on OS **(A)** and CSS **(B)** after PSM in the entire cohort. M, mastectomy; ABR, autologous breast reconstruction; OS, overall survival; CSS, cancer specific survival.

After PSM the 5-and 8-year OS rates were 93.7% and 91.6% for ABR group compared with 92.0% and 89.6% for mastectomy group. The 5-and 8-year CSS rates were 94.5% and 92.8% for patients undergoing ABR, as compared with 93.6% and 92.1% for patients undergoing mastectomy (**Table 2**). After PSM Kaplan–Meier survival analysis (**Figure 2**) showed ABR group had better OS (*P =* 0.001), but similar CSS (*P* = 0.174) between ARB and mastectomy groups. On univariate cox regression analysis, ABR still exerted a significant influence on the OS (HR, 0.82; 95%CI 0.72-0.92, *P*< 0.05). However, no statistical difference was shown on CSS (HR, 0.91; 95%CI 0.80-1.04, *P* = 0.17). On multivariate cox regression analysis, ABR was also associated with better OS (HR, 0.83; 95%CI 0.74-0.94, *P*< 0.05) and no statistical difference was shown on CSS (HR, 0.93; 95%CI 0.82-1.07, *P* = 0.31) (**Table 3**).

**Figure 3:**
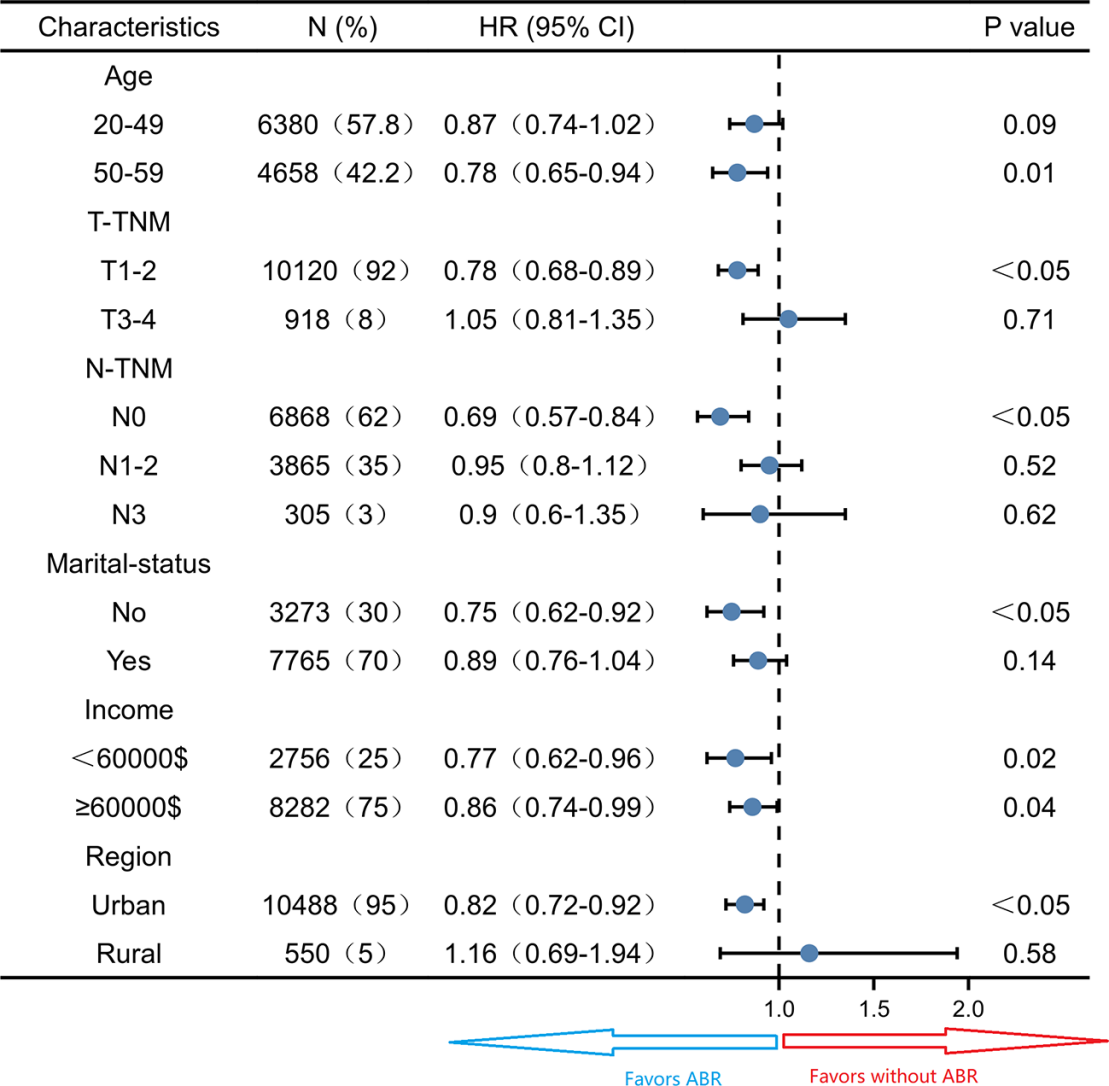
Subgroup analysis of overall survival in propensity score matched cohort (N = 11038).

**Figure 4:**
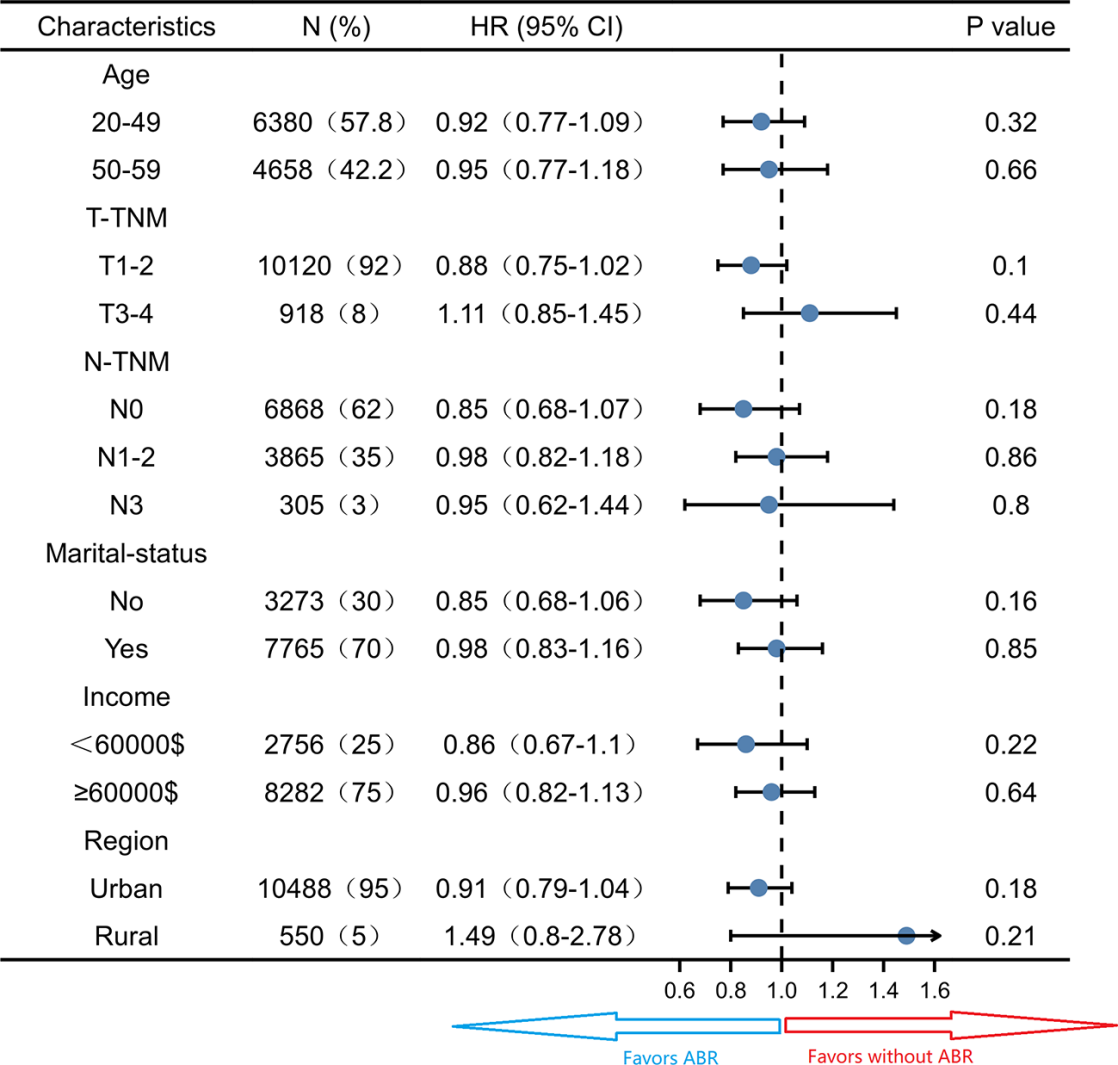
Subgroup analysis of cancer specific survival in propensity score matched cohort (N = 11038).

Subgroup survival analysis of the OS and CSS was performed stratified by the clinicopathological features after PSM. For OS, subgroup analysis suggested that ABR was favorable in patients with 50-59 years old (HR 0.78; 95% CI 0.65-0.94, *P* = 0.01) small tumor size (HR 0.78; 95% CI 0.68-0.89, *P*< 0.05), N0-TNM stage (HR 0.69; 95% CI 0.57-0.84, *P*< 0.05), no marriage partner (HR 0.75; 95% CI 0.62-0.92, *P*< 0.05) and living in urban (HR 0.82; 95% CI 0.72-0.92, *P*< 0.05). ABR was associated with better OS in both <60000$ (HR 0.77; 95% CI 0.62-0.96, *P =* 0.02) and ≥60000$ (HR 0.86; 95% CI 0.74-0.99, *P =* 0.04) income subgroups (**Figure 3**). For CSS, ABR was not shown to be associated with better CSS in any subgroups (**Figure 4**).

## Discussion

The number of women with breast cancer requesting reconstruction after surgery is increasing. Concerns have been raised regarding an increased risk of recurrence after breast reconstruction (20, 21). Following reconstruction by implant alone, no increased risk of recurrent disease has been shown (22–25). In the 1980s the latissimus dorsi (LD) flap was the method of choice, most often combined with a submuscular implant (26). Since 1994 most women have had breast reconstruction with a free microvascular transverse rectus abdominis muscle (TRAM) flap (27, 28), but this was superseded by the microvascular deep inferior epigastric artery perforator (DIEP) flap in 1998 (29). The long-term safety of ABR in BC patients remains controversial. Because previous research suggests that patients with breast cancer may harbour micrometastases at the time of reconstructive surgery. To our knowledge, our study represents one of the largest population-based studies to estimate the prognosis of ABR, offering a powerful insight into the role of ABR in female patients with BC. In the entire study, BC patients undergoing ABR after mastectomy had better long-term OS and CSS compared with that in patients undergoing mastectomy. After adjusting for baseline characteristics, analysis showed that ABR was associated with better OS, but this difference was not observed in CSS.

We use PSM to minimize the influence of clinicopathological variables on patient outcomes, and this is different from a series of previous studies (30, 31). Bezuhly M et al. (30) retrospectively analyzed CSS of 54660 BC patients undergoing mastectomy and breast reconstruction from 1998 to 2005 in the SEER database, finding that postmastectomy reconstruction is associated with improved breast CCS. But there was a significant imbalance in the baseline population in their data analysis with statistically significant differences, therefore the conclusions drawn may have some bias. As can be seen from our data that patients in the pre-matching ABR group have significantly lower later T-stage and N-stage. TNM stage is significantly related to the prognosis of patients. If the baseline matches are unbalanced, the accuracy of the results will be reduced. Although they have a large sample size, the year corresponding to its study population are far away from the present (1998–2005). Bezuhly M et al. (31) also concluded ABR carries no increased risk of breast cancer-specific mortality compared with mastectomy alone, however, the sample size in this study was far less than that in our current study, and there was no data related to OS at the end of the study.

Since Ben-Eliyahu S et al. (8) found that stress and surgical interventions promote cancer development by suppressing natural killer cell activity, many previous studies focused on the local recurrence rate and the risk of distant metastases after ABR. Although Isern, et al. (32) found a higher risk of recurrence with ABR after mastectomy, but more and more studies have found that autologous tissue reconstruction is safe and does not affect the local recurrence, distant metastasis and CSS of patients (18, 31, 33–35). A recent meta-analysis has reached the conclusion of delayed ABR leads to similar regional breast cancer recurrence rates compared to immediate ABR. But this study highlights the paucity of strong evidence on breast cancer recurrence after specific types and timings of ABR (36). Among these conclusions, identical to those reached in this article, is the analysis of CCS. It is possible that ABR may affect recurrence or distant metastases in patients in some specific cases. By our conclusion, it does not affect the long-term survival of patients, especially CSS. Therefore, according to our results, ABR is safe for post-mastectomy breast reconstruction, at least in terms of tumor-related factors that do not affect long-term survival.

Our data analysis concluded that ABR was associated with better OS, and possible reasons are unknown. It is possible that comorbidities can influence these results (37). Comorbidities such as cardiovascular disease, obesity, and smoking status were not evaluated in SEER database, and these conditions influence surgical treatment and long-term survival outcome. But the population we selected was young and middle-aged, with a relatively low risk of comorbidities. Of course, other psychosocial factors besides the patient’s disease that have an impact on the OS of patients. Previous studies have shown that income can affect the choice of surgical methods, and people with higher income are more likely to choose breast reconstruction (38). This can be clearly reflected in the data of this study before matching. We eliminated this bias as much as possible by using PSM. Some findings suggested that immediate reconstruction after mastectomy has a limited survival benefit when stratified according to household income. Autologous tissue reconstruction does not affect the survival outcome of patients (39). This is not consistent with the results of our subgroup analyses. The results of our subgroup analysis showed that patients had better OS in both <60000$ and ≥60000$ groups. However, for low-income patients, there are higher overall complications and infection rates after breast reconstruction (38). For the age factor, age ≥50 years was associated with increased breast drainage but did not seem to affect the success of breast reconstruction (40). In addition, our subgroup analysis showed that patients over 50 years of age may have better OS after ABR. Further research is needed to explore the reason. At the same time, the results of subgroup analysis shows that patients with earlier stage have better OS after ABR, possibly because these people are more likely to be cured and factors other than the disease have a greater impact on them than the disease itself. Similarly, marital status can also affect the choice of breast reconstruction (41), and patients with a marital partner have a higher rate of breast reconstruction, which is consistent with our results before matching. Subgroup analysis after matching showed that patients without a marital partner were more likely to benefit from ABR. We speculate that ABR may reduce their psychological trauma more, but further research is needed.

Social status is also an influencing factor for reconstruction. Women with lower socioeconomic status undergoing mastectomy were less likely to receive postmastectomy breast reconstruction (42). Although the SEER database does not provide detailed information, we do know the residence of patients at the time of disease, including urban or rural areas, which can indirectly reflect the social status of patients. People living in urban areas may have better social status and the proportion of people receiving ABR was higher in those living in urban areas before PSM. The results of subgroup analysis showed that OS was better for those receiving ABR who lived in urban areas. Some studies have shown that patients receiving ABR have higher education compared to mastectomy patients (43), and patients with commercial insurance and higher levels of education were more likely to undergo post-mastectomy reconstruction (44). These social factors can lead to a positive impact in OS, but it is difficult to evaluated this impact. While the number of BC cases is increasing, more and more patients will be cured as treatments continue to increase, meaning that more patients will face losing their breasts in later life. When patients are cured, it will no longer be the disease itself that affects OS, but other social and psychological aspects. Therefore, in future studies, we need to pay more attention to the psychosocial impact on patients. In conclusion, ABR after mastectomy was associated with better OS, but not affect CSS, which provides strong evidence that patients choose autologous tissue reconstruction after mastectomy.

Of course, our study has some limitations, because the SEER database does not give data on patients receiving endocrine therapy and targeted therapy, and our comparison cannot be matched to the relevant data, which are crucial for the treatment of the relevant subtypes of breast cancer. Also, the SEER database does not provide data on patient recurrence, which does not allow us to validate some previous studies and underlying theories. Confounding factors such as education, insurance, and comorbidities also could not be evaluated in this paper. Also, the impact of immediate reconstruction and late reconstruction was not evaluated. Immediate reconstruction is performed in better patients, and late reconstruction is performed in worse patients that survived, and this study do not evaluate this condition.

In summary, to our knowledge, for the first time, with PSM performing in a large sample size of ABR patients, that compared to receiving mastectomy patients. ABR does not negatively affect breast CCS, however, ABR is associated with better OS. This study lends further evidence for the oncologic safety of breast reconstruction.

## Data availability statement

The datasets presented in this study can be found in online repositories. The names of the repository/repositories and accession number(s) can be found in the article/Supplementary Material.

## Author contributions

YL and SW conceived and designed the idea to this paper. SW and XM organized the data. SW analyzed the data and drafted the paper. XZ and CY analyzed the data. YW revised the final paper. All authors contributed to the article and approved the submitted version.
